# Neddylation of EphB1 Regulates Its Activity and Associates with Liver Fibrosis

**DOI:** 10.3390/ijms24043415

**Published:** 2023-02-08

**Authors:** Rongxin Li, Dan Zhang, Yueqing Han, Ke Chen, Weiran Guo, Yijun Chen, Shuzhen Wang

**Affiliations:** School of Life Science and Technology, China Pharmaceutical University, Nanjing 211198, China

**Keywords:** liver fibrosis, hepatic stellate cells, EphB1, neddylation, ubiquitination, MLN4924

## Abstract

Liver fibrosis is a pathological process characterized by the excessive synthesis and accumulation of extracellular matrix proteins (ECMs) contributed mainly by the activated hepatic stellate cells (HSCs). Currently, no direct and effective anti-fibrotic agents have been approved for clinical use worldwide. Although the dysregulation of Eph receptor tyrosine kinase EphB2 has been reported to associate with the development of liver fibrosis, the involvement of other Eph family members in liver fibrosis remains underexplored. In this study, we found that the expression of EphB1 is significantly increased accompanying remarkable neddylation in activated HSCs. Mechanistically, this neddylation enhanced the kinase activity of EphB1 by the prevention of its degradation, thereby promoting the proliferation, migration, and activation of HSCs. Our findings revealed the involvement of EphB1 in the development of liver fibrosis through its neddylation, which provides new insights into the Eph receptor signaling and a potential target for the treatment of liver fibrosis.

## 1. Introduction

Liver fibrosis is the consequences of a dynamic wound-healing process in response to chronic liver injury, which is characterized by the deposition of extracellular matrix proteins (ECMs) [1]. In this process, activated hepatic stellate cells (HSCs) play a central role by producing excessive ECMs and releasing inflammatory factors [2]. Liver fibrosis is also a reversible process, and usually reversed after elimination of damage factors. However, ongoing liver injury can result in the development of liver fibrosis to cirrhosis; once cirrhosis is established, the possibility of reversing the process is reduced and complications occur [3]. Therefore, liver fibrosis plays a key role in the progression of chronic liver diseases, and patients with liver fibrosis are at significant risk of developing hepatocellular carcinoma (HCC). Although much great progress has been achieved in understanding the pathogenesis and clinical consequences of liver fibrosis, no antifibrotic drug has been approved [4]. Therefore, there is an exigent need to dissect more of the molecular mechanism underlining liver fibrosis in order to discover novel pharmacological strategies to alleviate or reverse liver fibrosis.

Abnormal expression of tyrosine kinase is associated with many diseases, and has become important targets for treatment of liver fibrosis [5,6]. Eph receptors are the largest family of receptor tyrosine kinases and their abnormal expression were originally discovered in human carcinoma [7]. Their involvement in many other pathological conditions, such as tissue fibrosis, is increasingly recognized [8]. We and others have previously reported that increased expression of EphB2 is associated with liver fibrosis, and the deficiency of EphB2 by knockout or microRNA can alleviate the development of liver fibrosis [9,10,11]. However, other members of Eph receptors that contribute to the progression of liver fibrosis have yet to be fully identified.

EphB1, a member of the Eph family, plays a functional role in synapse formation and maturation, angiogenesis, development of immune organs, and migration and invasion of cancer cells [12,13]. In addition, EphB1 has been identified as one of the seven commonly upregulated genes in all stages of HCC by bioinformatics analysis of the data containing 371 primary HCC tumor samples and 50 adjacent normal liver tissue samples [14]. This study also reported that high expression levels of EphB1 are correlated with poor overall survival of HCC patient. A common strategy is usually used to screen out genes linked to the development of liver diseases by using models from hepatitis to HCC transformation. We believe that the reverse might sometimes also be feasible. Given the important roles of Eph receptors in tissue fibrosis and the high expression of EphB1 in HCC, we therefore postulated that EphB1 would also contribute to the progression of liver fibrosis.

In this study, we investigated the expression and roles of EphB1 in HSCs and the mouse model of liver fibrosis. We also revealed a novel post-translational modification (PTM), namely neddylation, of EphB1 in activated HSCs. Neddylation is one of the PTMs of proteins that covalently attaches the ubiquitin (Ub)-like molecule NEDD8 (neural precursor cell expressed developmentally downregulated protein 8) to the lysine residues of target proteins, modifying their biochemical properties and three-dimensional structures [15]. Like ubiquitination, the process of neddylation is sequentially catalyzed by NEDD8-activating enzyme (NAE1), conjugating enzyme (E2) and E3 ligases, which can be reversed by proteases such as NEDD8-Specific Protease 1 (NEDP1) and JAB1/CSN5 [16,17]. Herein, we present that EphB1 neddylation stabilizes its protein level and increases its phosphorylation during the activation of HSCs. Hence, the inhibition of EphB1 and its neddylation could be a potential therapeutic approach for the treatment of liver fibrosis.

## 2. Results

### 2.1. EphB1 Expression Is Increased in Activated HSCs

**Figure 1:**
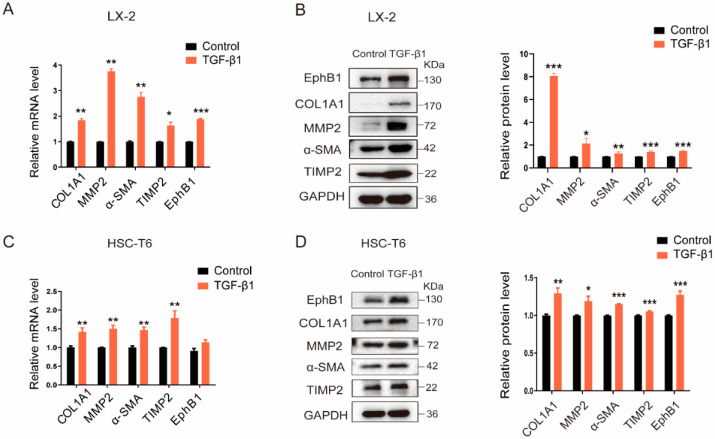
EphB1 expression is increased in activated HSCs. (**A**,**B**) The relative mRNA and protein levels of EphB1 and the typical pro-fibrotic marker proteins in human LX-2 cells stimulated with or without TGF-β1 (5 ng/mL). (**C**,**D**) The relative mRNA and protein levels of EphB1 and several other typical pro-fibrotic genes in rat HSC-T6 cells stimulated with or without TGF-β1 (5 ng/mL). Values shown are means ± SEM: * *p* < 0.05, ** *p* < 0.01, *** *p* < 0.001 vs. control.

To determine whether EphB1 takes part in the activation of HSCs, we examined its expression in both human and rat HSCs activated by transforming growth factors β1 (TGF-β1), one of the best-known pro-fibrotic cytokines. The results of RT-PCR and western blotting showed that both the mRNA and protein levels of EphB1 and the typical pro-fibrotic markers, such as COL1A1, MMP-2, α-SMA, and TIMP2, were all significantly upregulated by TGF-β1 stimulation in LX-2 cells (Figure 1A,B). Consistently, similar data were also obtained in the activation process of the rat HSC-T6 cells (Figure 1C,D). These results demonstrated that EphB1 expression is significantly increased in the activated HSCs, suggesting that there is a potential connection between EphB1 and the activation of HSCs.

### 2.2. EphB1 Undergoes Augmented Neddylation in Activated HSCs

**Figure 2:**
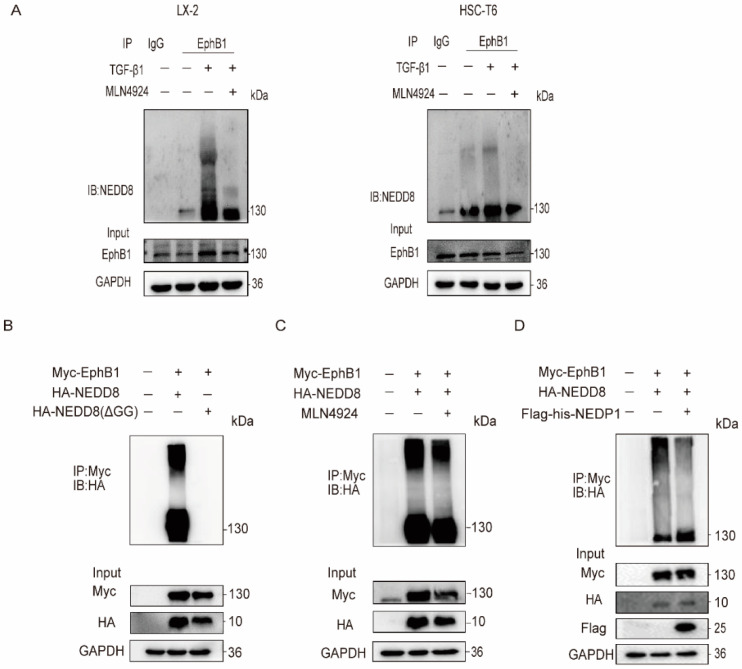
EphB1 is neddylated in activated HSCs. (**A**) Analysis of EphB1 neddylation in HSCs treated with or without TGF-β1 (5 ng/mL) and MLN4924 (1 μM) for 24 h. (**B**) HA-immunoblot of Myc-immunoprecipitate from HEK293T cells transfected with Myc-tagged EphB1 and HA-tagged NEDD8 or NEDD8 ΔGG. (**C**) Effects of MLN4924 on the EphB1 neddylation in HEK293T cells transfected with the indicated constructs. (**D**) Effects of NEDP1 on the EphB1 neddylation in HEK293T cells transfected with the indicated constructs.

In view of the overactivation of neddylation pathway that has been described during liver fibrosis [18], we were inspired to explore whether EphB1 could be conjugated to NEDD8 in HSCs. EphB1 was immunoprecipitated from the whole lysates of LX-2 cells with an antibody specifically reacts to EphB1, and then subjected to western blotting with an anti-NEDD8 antibody. The results revealed that EphB1 was indeed neddylated by NEDD8 in HSCs, and its neddylation levels were markedly enhanced by TGF-β1 stimulation and inhibited by the small molecular inhibitor of NAE1, MLN4924 [19] (Figure 2A). Of note, neddylation modification was not observed in two other tested members of EphB family in HSCs including EphB2 and EphB3, suggesting a special activity regulation mode of EphB1. To further validate our findings, we co-transfected exogenous Myc-tagged EphB1 and HA-tagged NEDD8 or NEDD8 ΔGG (deletion of the Gly-75/76 residues of NEDD8 can abolish its covalent conjugating ability [20]) in HEK293T cells. The immunoprecipitation results showed that EphB1 could be neddylated by NEDD8, rather than the conjugation-defective NEDD8 ΔGG (Figure 2B). Meanwhile, MLN4924 treatment (Figure 2C) or the co-expression of the de-neddylation enzyme NEDP1 (Figure 2D) greatly reduced the neddylation of EphB1. Collectively, these results showed that EphB1 can be neddylated, and that its neddylation is augmented in activated HSCs.

### 2.3. EphB1 Neddylation Enhances Its Kinase Activity and Inhibits Its Degradation

**Figure 3:**
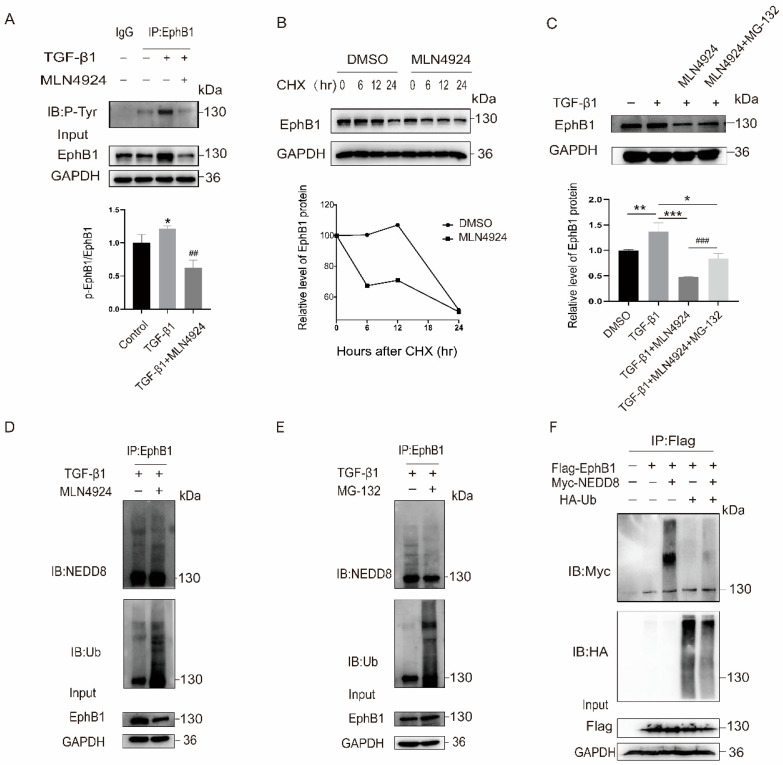
Neddylation stabilizes EphB1 by antagonizing its ubiquitination. (**A**) Effects of neddylation blockade by MLN4924 (1 μM) on the EphB1 phosphorylation in LX-2 cells. * *p* < 0.05 vs. Control; ## *p* < 0.01 vs. TGF-β1 treatment group. (**B**) Assessment of EphB1 protein stability in LX-2 cells. LX-2 cells were treated with DMSO or MLN4924 (1 μM) for 24 h, and then with CHX (100 μg/mL) for various periods of time as indicated. Cell lysates were then harvested and subjected to immunoblotting with the indicated antibodies. (**C**) EphB1 protein expression in LX-2 cells treated with MLN4924 (1 μM), MG-132 (10 μM), or their combination. Values shown are means ± SEM. * *p* < 0.05, ** *p* < 0.01, *** *p* < 0.001 vs. DMSO; ### *p* < 0.001 vs. MLN4924 treatment group. (**D**) Analysis of EphB1 neddylation and ubiquitination levels in activated LX-2 cells treated with or without MLN4924 (1 μM). (**E**) Analysis of EphB1 neddylation and ubiquitination levels in activated LX-2 cells treated with or without MG-132 (10 μM). (**F**) Analysis of EphB1 neddylation and ubiquitination levels in HEK293T cells transfected with flag-tagged EphB1, HA-tagged Ub, or Myc-tagged NEDD8 as indicated.

Given that neddylation pathway plays a significant role in the regulation of protein function and stability, we next investigated the effect of EphB1 neddylation on its kinase activity and protein stability. The results showed that EphB1 neddylation varied as the same trend with its phosphorylation and kinase activation of EphB1, and neddylation blockade by MLN4924 resulted in decreased EphB1 phosphorylation (Figure 3A), implying neddylation might contribute to the tyrosine kinase activity of EphB1. Given that neddylation inhibition by MLN4924 led to a decrease in the overall protein expression of EphB1, we next explored the effect of neddylation on the protein stability of EphB1 by using protein synthesis inhibitor cycloheximide (CHX). The results showed that MLN4924 treatment shortened the half-life of endogenous EphB1 (Figure 3B), indicating that neddylation of EphB1 might promotes its stabilization. To investigate the role of ubiquitination-mediated proteasomal pathway in the EphB1 degradation, the activated LX-2 cells were treated with proteasome inhibitor MG-132. Following immunoblotting, analysis revealed that cotreatment of MG-132 with MLN4924 significantly augmented the protein level of EphB1 compared to MLN4924-alone treated cells (Figure 3C). We then detected whether the enhanced stability of neddylated EphB1 was possibly the result of reduced protein degradation. Indeed, inhibition of neddylation pathway by MLN4924 enhanced EphB1 ubiquitination levels (Figure 3D), while MG132-mediated inhibition of the ubiquitin–proteasome pathway inhibited EphB1 neddylation (Figure 3E). The inverse correlation of EphB1 neddylation to its ubiquitination levels was also evidenced in HEK293T cells transfected with NEDD8 and ubiquitin (Ub) (Figure 3F). Thus, EphB1 neddylation contributes to its kinase activity, attenuates its ubiquitination, and inhibits its degradation.

### 2.4. EphB1 Promotes the Proliferation, Migration, and Activation of HSCs

**Figure 4:**
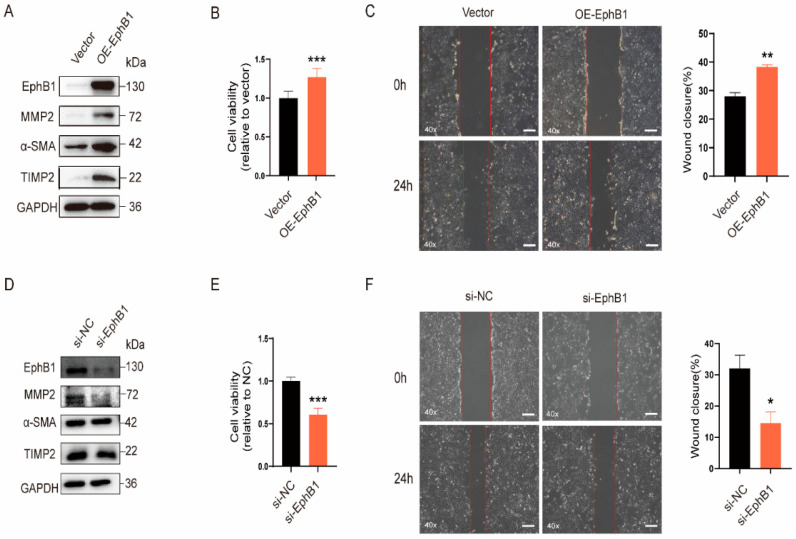
EphB1 promotes the proliferation, migration, and activation of HSCs. (**A**) Effects of exogenous overexpression of EphB1 on the expression of pro-fibrotic markers in LX-2 cells. (**B**) Effects of EphB1 overexpression on the cell proliferation of LX-2 cells. (**C**) Microscopic inspection of LX-2 cells transfected with empty vector or EphB1 expression plasmid after scratching (0 h) and after 24 h of wound healing (40× magnification, scale bar, 200 μm). (**D**) Effects of EphB1 knockdown on the expression of pro-fibrotic markers in LX-2 cells. (**E**) Effects of EphB1 knockdown on the cell proliferation of LX-2 cells. (**F**) Effects of EphB1 knockdown on the migration of LX-2 cells as determined in (**C**) (40× magnification, scale bar, 200 μm). Values shown are means ± SEM. * *p* < 0.05, ** *p* < 0.01, *** *p* < 0.001 vs. control.

To elucidate the role of EphB1 in the activation of HSCs, the effect of overexpression or knockdown of EphB1 on the proliferation, migration, and activation of HSCs was examined. As shown in Figure 4, exogenous overexpression of EphB1 markedly increased the expression of the pro-fibrotic marker proteins (Figure 4A), and promoted the proliferation (Figure 4B) and migratory capacity (Figure 4C) of LX-2 cells. Meanwhile, the knockdown of EphB1 by small interfering RNA (siRNA) manifested the opposite effects and reversed the pro-fibrotic effects of TGF-β1 (Figure 4D–F). These findings indicated that EphB1 plays a pro-fibrotic role in liver fibrogenesis.

### 2.5. EphB1 Expression and Its Neddylation Elevated in CCl_4_-Induced Liver Fibrosis Mice

**Figure 5:**
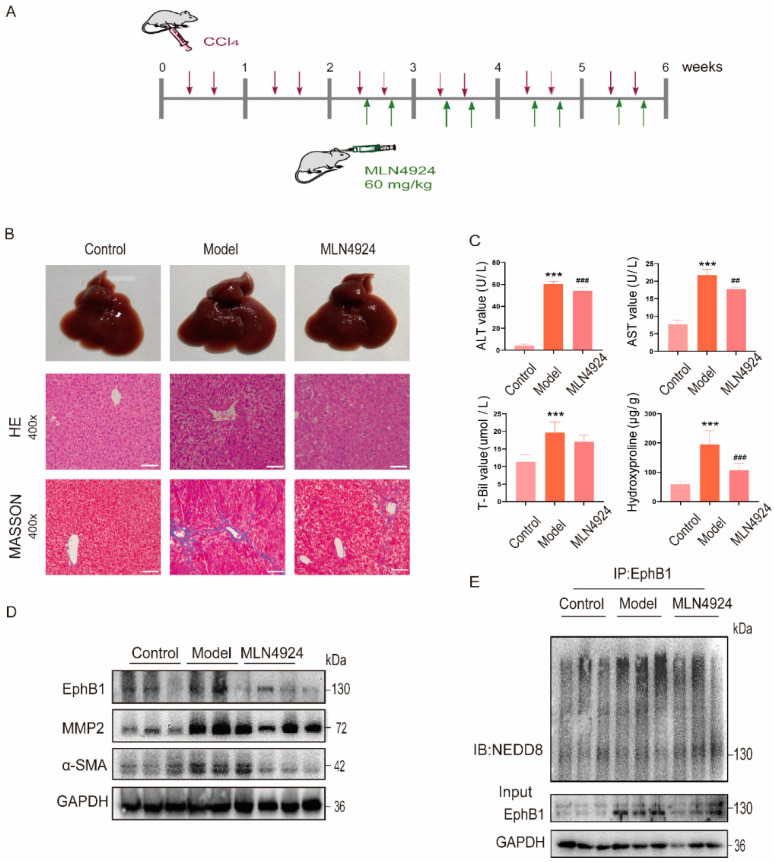
EphB1 is neddylated in CCl_4_-induced liver fibrosis mice. (**A**) Schema of the mice experimental protocol. Mice were administrated as indicated by the arrows. (**B**) Representative histopathological images of H&E and Masson’s trichrome staining of mouse liver sections (400× magnification, scale bar, 100 μm). (**C**) Serum levels of ALT, AST, T-Bil, and hydroxyproline content in mouse livers. (**D**) The protein expression of EphB1 and the pro-fibrotic marker proteins in the liver tissues of mice treated with Oil (Control group), CCl_4_ (Model group), CCl_4,_ plus MLN4924 (MLN4924 group). (**E**) Analysis of EphB1 neddylation in the liver tissues of mice treated with Oil, CCl_4_, CCl_4_ plus MLN4924. Values shown are means ± SEM. *** *p* < 0.001 vs. Control; ## *p* < 0.01, ### *p* < 0.001 vs. Model.

To investigate the role of EphB1 in the pathogenesis of liver fibrosis, we examined its expression and post-translational modification in CCl_4_-induced mouse model of liver fibrosis (Figure 5A). Hematoxylin and eosin (H&E) and Masson staining of the liver sections revealed that mice of the CCl_4_ treatment group manifested characteristic fibrotic phenotypes, such as collagen deposition and ballooning (Figure 5B). Correspondingly, the levels of serum alanine transaminase (ALT), aspartate transaminase (AST), total bilirubin (T-bil), and hydroxyproline were also significantly higher in model mice than in the corresponding controls (Figure 5C), while neddylation inhibition by MLN4924 remarkably alleviates CCl_4_-induced liver fibrosis (Figure 5B,C). In line with this, the protein levels of EphB1 and the pro-fibrotic marker proteins showed sharp reduction in liver tissues after MLN4924 treatment (Figure 5D). Moreover, the neddylation of EphB1 was greatly enhanced in CCl_4_-induced mice and dramatically reduced after MLN4924 treatment (Figure 5E). Above all, our results suggest that neddylation modification of EphB1 enhances its kinase activity and protein stability, thereby contributing to the pathogenesis of liver fibrosis.

## 3. Discussion

Protein PTMs such as phosphorylation, acetylation, glycosylation, hydroxylation, ubiquitination, neddylation, and many others are considered as small molecules covalently bind to the target proteins for dynamic control of protein function after protein synthesis. Abnormal PTMs and related molecules are increasingly implicated in the pathogenesis of liver-related diseases [21,22]. For example, the presence of immunoreactive ubiquitin in liver biopsy specimens has been suggested as an early marker for cell injury in nonalcoholic steatohepatitis (NASH) [23]. Consistently, protein expression of deubiquitinase ubiquitin C-terminal hydrolase 1 (UCHL1) is highly induced upon HSC activation, and UCHL1 pharmacological inhibition ameliorates liver fibrogenesis in mice. [24]. The role of SUMOylation in liver fibrosis has been reported in that coadministration of SUMOylation inhibitors increased the efficacy of farnesoid X receptor agonists against HSC activation and fibrosis [25]. In addition to ubiquitination and SUMOylation, recent studies have also demonstrated the overactivation of the neddylation pathway in various aspects and phases of chronic liver diseases [26]. Multiple substrates of neddylation have been uncovered such as transcriptional regulators (c-Jun, NRF2, IkBa, HIF1a/HIF2a, and SRSF3) and proteins involved in signaling pathway transduction and lipid metabolism (AKT, LKB1, TGFβRII, ETFs, and SREBP1c) in hepatic dysfunctions [26]. In the present study, EphB1 was disclosed and validated as a novel target of neddylation in both activated HSCs and the CCl_4_-induced mouse model, which expands further the endogenous protein substrate spectrum of neddylation in liver fibrosis.

Compelling evidence has shown that different PTMs can function together to affect protein homeostasis, activity, subcellular localization, and interactions by inducing functional and structural changes of the target proteins [27,28]. For example, neddylation has been reported to limit basal MKK7 kinase activity in breast cancer cells [29], and to regulate LKB1 and Akt stabilization in liver cancer cells [30]. Given that phosphorylation and ubiquitination are two mostly investigated PTMs, their potential relevance with neddylation was then explored to understand the underlying mechanisms regulating EphB1 kinase activity and stability in this study. Our data showed that neddylation inhibition reduced the phosphorylation level of EphB1, suggesting a synergistic effect between EphB1 neddylation and phosphorylation (active form of EphB1). On the other hand, neddylation can function antagonistically with ubiquitination and stabilize the protein level of EphB1. However, whether these PTMs of EphB1 are linked through conformational changes still needs further study.

Due to certain homology of the Eph family, there are some different degrees of similarities in their functions. For example, it has been reported that EphB1, EphB2, and EphB3 all participated in the morphogenesis and synaptic formation of dendritic spines in the hippocampus [31]. The role of EphB2 in promoting HSCs activation and liver fibrosis progression has been well documented [9,10,11]. In present study, similar pro-fibrotic effects of EphB1 were demonstrated by gene overexpression and knockdown. However, our preliminary study showed that EphB1 was the only NEDD8-conjugated protein among EphB1 to EphB3 in activated HSCs, suggesting that the activation of EphB1 and the way it functions may be different from EphB2 in liver fibrosis. It has been shown that some Eph receptors can be phosphorylated by other Eph receptors through reciprocal regulation [32], and whether EphB1 and EphB2 would synergistically contribute to liver fibrosis remains to be explored.

Despite the similar three-step enzymatic reactions of neddylation process to ubiquitination, a far smaller number of NEDD8 E3 ligases than E3 ubiquitin ligases were identified [33]. Interestingly, some known ubiquitin E3 ligases can also play a role as NEDD8 E3 ligases in protein PTMs. For example, the well-known ubiquitin E3 ligase C-CBL could mediate the neddylation of EGFR [34], TβRII [35], and c-Src [36] in different cancer cells. In view of that C-CBL was previously demonstrated to induce EphB1 ubiquitination and degradation in Chinese hamster ovary (CHO)-EphB1 cells [37], we investigated the possibility of C-CBL as the NEDD8 E3 ligase of EphB1 in HSCs. However, our efforts revealed that C-CBL mainly functions as ubiquitin E3 ligase of EphB1 to promote its ubiquitination, rather than neddylation, suggesting that there is an unknown E3 ligase for EphB1 neddylation in HSCs.

Although the mechanisms that activate neddylation pathway in liver fibrosis are not exactly understood, a reduction in the liver damage through neddylation inhibition by MLN4924 treatment has been described in different mouse models of liver fibrosis [19]. In agreement with these studies, our data confirmed that MLN4924 effectively ameliorates CCl_4_-induced liver fibrosis at least partly through inhibiting EphB1 neddylation. Taken together, our study defined neddylation as a novel modification of EphB1 and an association between EphB1 neddylation and liver fibrosis.

## 4. Materials and Methods

### 4.1. Reagents

TGF-β1 was purchased from R&D Systems (Minneapolis, MN, USA). MLN4924 was purchased from MedChemExpress (Monmouth Junction, NJ, USA) and dissolved in dimethyl sulfoxide (DMSO). Mouse anti-EphB1 (sc-130054) was obtained from Santa Cruz Biotechnology (Dallas, TX, USA) and anti-p-Tyr (4G10, #05-321X) was purchased from Millipore (Billerica, MA, USA). Antibodies against NEDD8 (2745S), HA (3724S), TIMP2 (5738S) and Ub (3936T) were purchased from Cell Signaling Technology (Danvers, MA, USA). Antibodies against MMP2 (A6247), α-SMA (A1011), COL1A1 (A1352) and Myc-tag (AE070) were purchased from Abclonal (Wuhan, China). The HRP-conjugated anti-rabbit (7074S) and anti-mouse (7076S) antibodies were from Cell Signaling Technology (Danvers, MA, USA). Anti-GAPDH (60004-1-Ig) was purchased from Proteintech (Wuhan, China).

### 4.2. Cell Lines and Culture

The human LX-2 and rat HSC-T6 cells were purchased from Procell Life Science & Technology (Wuhan, China) and the Chinese Academy of Sciences Shanghai Institute of Cell Bank (Shanghai, China), respectively. Human embryonic kidney cells (HEK293T) cells were obtained from KeyGEN Biotech (Nanjing, China). All cell lines were cultured in Dulbecco’s modified Eagle’s medium (DMEM) with 10% fetal bovine serum (FBS) (TransGen Biotech, China) and incubated at 37 °C in a humidified incubator in 5% CO_2_.

### 4.3. Animal Models

C57BL/6J mice were acquired from Hangzhou Ziyuan Laboratory Animal Technology Co. Ltd. After adaptively feeding for one week, the mice were randomly divided into three groups: control group (*n* = 6), model group (*n* = 6), and treatment group (*n* = 6). Mice from control group were injected intraperitoneally with corn oil at a dose of 10 mL/kg twice a week. To establish the liver fibrosis model, CCl_4_ (Sigma, St. Louis, MO, USA) was administered by intraperitoneal injection at a dose of 10 mL/kg twice a week for six weeks [11]. Two weeks after the first CCl_4_ administration, CCl_4_-induced mice were treated with MLN4924 for four weeks by subcutaneous injection at a dose of 60 mg/kg twice a week [18,19]. All mice were sacrificed 48 h after the last injection and the serum and liver tissues were collected for subsequent analysis. All the animal experiments were approved by the Animal Experimentation Ethics Committee of China Pharmaceutical University.

### 4.4. Plasmids, siRNAs and Transfection

The mRNA sequences coding EphB1, NEDD8, NEDP1, and Ub were amplified and inserted into pcDNA3.1 vector, respectively. HA-NEDD8 (ΔGG) is a mutant NEDD8 that cannot be conjugated with the target substrates. SiRNA was synthesized by Biomics (Nantong, China). The sequence of siRNA targeting EphB1(si-EphB1) was 5′-GGATGAAGATCTACATTGA-3′ and provided by RiboBio (Guangzhou, China). HEK293T cells were plated in 10 cm cell culture dishes and transfected with 8 μg of the indicated plasmids by using Lipofectamine 2000 (Invitrogen, Waltham, MA, USA). After 48 h, cells were used for western blot assays.

### 4.5. Western Blotting

Proteins were extracted from the mouse liver tissues or cultured cells and quantified using BCA Protein Assay Kit (Generay Biotech, Shanghai, China). Then, the samples were separated by SDS-PAGE and transferred to PVDF membrane (Millipore, Billerica, MA, USA). After blocking with 5% skim milk for 2 h, the membranes were incubated with primary antibodies overnight at 4 °C, and then probed with HRP-conjugated secondary antibodies for 2 h at room temperature. The signals were finally detected with ECL Kit.

### 4.6. Immunoprecipitation

For immunoprecipitation assays, cell lysates were centrifuged, quantified, and incubated with the appropriate primary antibody and protein A agarose beads overnight at 4 °C. Then, the beads were washed with lysis buffer three times. After washing, the beads were boiled with SDS loading buffer at 100 °C for 5 min for subsequent western blot analysis.

### 4.7. Quantitative RT-PCR (qRT-PCR)

The total RNAs were extracted from cells and liver tissues by using TRIzol reagent (Invitrogen, Carlsbad, CA, USA), and the concentrations were detected by Nanodrop. Subsequently, the reverse transcription was conducted with PrimeScript™ RT Master Mix Kit and RT-qPCR was performed using SYBR Green Master Mix (Vazyme Biotech, Nanjing, China). The relative mRNA level of all genes was normalized to that of GAPDH. The primers used are listed in Table S1.

### 4.8. Biochemical Assays

After obtaining mouse blood samples, the serum level of ALT, AST and total T-bil were measured by Alanine Aminotransferase Assay Kit, Aspartate Aminotransferase Assay Kit, and Total Bilirubin Assay Kit (Institute of Biological Engineering, Nanjing, China), respectively. The content of hydroxyproline in mouse liver was determined according to the manufacturer’s protocol of Hydroxyproline Testing Kit (Nanjing Jiancheng Bioengineering Institute, Nanjing, China).

### 4.9. H&E and Masson’s Trichrome Staining

The liver tissues of mice were fixed with 4% paraformaldehyde. Paraffin sections were prepared and stained with H&E staining and Masson’s trichrome Staining Kit (Servicebio Technology, Wuhan, China).

### 4.10. Cell Proliferation Assay

LX-2 cells were transfected with empty vector or EphB1 expression plasmid for 24 h before suspending in culture medium and inoculated in a 96-well plate. After 24 h of being treated with 5 ng/mL TGF-β1, 10 μL CCK-8 solution (Dojindo, Shanghai, China) was added to each well. The plate was incubated for 1.5 h before measuring the optical density (OD) value at 450 nm using a microplate reader (Bio-Tek, Winooski, VT, USA).

### 4.11. Wound Healing Assay

LX-2 cells were transfected with empty vector or EphB1 expression plasmid and cultured in six-well plate until the confluence reached 70%. Then, the cells were scraped by a pipette tip, each well was washed with PBS, and cells were treated with TGF-β1 for 24 h. Images for scratch were observed at 0 h and 24 h after scratching. The wound closure was calculated by using the following formula:

$$Wound\;closure\;\%=\frac{Area\;of\;the\;wound\;at\;0\;h-Area\;of\;the\;wound\;at\;24\;h}{Area\;of\;the\;wound\;at\;0\;h}\times 100\;\%$$

### 4.12. Quantification and Statistical Analysis

All data were expressed as the mean ± SEM. Statistical analyses were carried out with GraphPad Prism 9.0 (GraphPad software Inc., San Diego, CA, USA) using unpaired two-tailed Student’s *t*-test. Differences were considered to be significant for values of *p* < 0.05.

## Data Availability

All data is contained within the article and Supplementary Materials.

## Supplementary Materials

The following supporting information can be downloaded at: https://www.mdpi.com/article/10.3390/ijms24043415/s1.
