# Supplementary material for: Neddylation of EphB1 Regulates Its Activity and Associates with Liver Fibrosis
Source: Int J Mol Sci. 2023 Feb 8;24(4):3415. doi: 10.3390/ijms24043415 (PMC9964663; doi:10.3390/ijms24043415)
Supplement: Supplementary file 1 [file ijms-24-03415-s001.zip › ijms-2181475-supplementary.pdf]

Table S1. List of all primers used in this study.

Genes name	Forward Primers	Reverse Primers
EphB1 (human)	5′-TGTGAGATGGACAGCTCCAGAG-3′	5′-TGCCACAGTCTTGAGACTTGCC-3
α-SMA (human)	5′-GGGAATGGGACAAAAAGACA-3′	5′-GGGAATGGGACAAAAAGACA-3′
COL1A1 (human)	5′-CTTCAGGGGCAACACGAA-3′	5′-GGAACACCTCGCTCTCCA-3′
MMP-2(human)	5′-GTATTTGATGGCATCGCTCA-3'	5′-CATTCCCTGCAAAGAACACA-3′
TIMP2(human)	5′-GTCACAGAGAAGAACATCAACG-3′	5′-GATGTCGAGAAACTCCTGCTT-3′
GAPDH(human)	5′-GGAGCGAGATCCCTCCAAAAT-3'	5′-GGCTGTTGTCATACTTCTCATGG-3′
EphB1 (rat)	5′-CTTTGGCCTCTCTCGCTACC-3'	5′-ATCGCTGGCTGACGTAAACT-3'
α-SMA (rat)	5′-TGTGCTGGACTCTGGAGATG-3'	5′-GAAGGAATAGCCACGCTCAG-3'
COL1A1 (rat)	5′-ATGTTCAGCTTTGTGGACCT-3'	5′-CAGCTGACTTCAGGGATGT-3'
MMP-2(rat)	5′-GTGACGGCTTCCTCTGGTGTTC-3'	5′-CAGGGCTGTCCATCTCCATTGC-3'
TIMP2(rat)	5′-TGATGCTAAGCGTGTCCCAG-3'	5′-GCTGGACGTTGGAGGAAAGA-3'
GAPDH(rat)	5′-GAACCTGCCGTGGGTAGAG-3'	5′-AGGTCGGTGTGAACGGATTTG-3'
